# Supplementary material for: Frailty trajectory and its associated factors in older patients undergoing abdominal surgery involving the digestive system: A longitudinal study
Source: PLoS One. 2025 Aug 8;20(8):e0330093. doi: 10.1371/journal.pone.0330093 (PMC12334016; doi:10.1371/journal.pone.0330093)
Supplement: S1 Data — (DOCX) [file pone.0330093.s001.docx]

表1 纳入研究对象的基线资料

| 变量 | 分类 | 全部纳入患者（n=155） | 基线无衰弱患者（n=58） | 基线衰弱前期患者（n=64） | 基线衰弱患者（n=33） | X^2^ | P |
| --- | --- | --- | --- | --- | --- | --- | --- |
|  |  | 频数（%） | 频数（%） | 频数（%） | 频数（%） |  |  |
| 性别 | 男 | 76（49） | 30(50.8) | 31（48.4） | 15(45.5) | 31.375 | 0.965 |
|  | 女 | 79（51） | 28(49.2) | 33（51.6） | 17(51.5) |  |  |
| 年龄 | ＜65岁 | 42（27.2） | 14(24.6) | 22（34.4） | 6(18.2) | 10.456 | 0.097^*^ |
|  | 65—74岁 | 71（45.8） | 29(50) | 26（40.6） | 16(48.5) |  |  |
|  | 75—84岁 | 35（22.6） | 11(19.7) | 15（23.4） | 9(27.3) |  |  |
|  | ≥85岁 | 7（4.5） | 4(5.7) | 1（1.6） | 2(6.1) |  |  |
| BMI（kg/m^2^） | ＜18.5 | 5（3.2） | 2(3.3) | 3（4.7） | 0 | 11.236 | 0.107 |
|  | 18.5—23.9 | 67（43.2） | 31(54.1) | 23（35.9） | 13(39.4) |  |  |
|  | 24—27.9 | 44（28.4） | 14(24.6) | 19（29.7） | 11(33.3) |  |  |
|  | ≥28 | 39（25.2） | 11(18) | 19（29.7） | 9(27.3) |  |  |
| 吸烟史 | 无 | 117（75.5） | 44(75.4) | 49（76.6） | 24(72.7) | 33.041 | 0.979^*^ |
|  | 有 | 18（11.6） | 10(12.3) | 1（1.6） | 7(21.2) |  |  |
|  | 曾经吸烟 | 18（11.6) | 4(12.3) | 12（18.8） | 2(6.1) |  |  |
| 饮酒史 | 无 | 113（72.9） | 42(73) | 46（71.9） | 25(75.8) | 30.842 | 0.886^*^ |
|  | 有 | 31（20） | 12(19.7) | 13（20.3） | 6(18.2) |  |  |
|  | 曾经饮酒 | 11（7.1） | 4(7.4) | 5（7.8） | 2(6.1) |  |  |
| 居住地 | 城镇 | 124（80） | 47(80.3) | 55（85.9） | 22(66.7) | 32.511 | 0.973 |
|  | 农村 | 31（20） | 11(19.7) | 9（14.1） | 11(33.3) |  |  |
| 居住状况 | 独居 | 9（5.8） | 1(1.72) | 3（4.7） | 6(45.4) | 8.125 | 0.069 |
|  | 与配偶居住 | 112（72.3） | 45(77.6） | 58（90.6） | 9(27.3) |  |  |
|  | 与子女居住 | 34（21.9） | 12(20.7) | 13（20.3） | 9(27.3) |  |  |
| 婚姻状况 | 已婚 | 130（83.9） | 49(83.6) | 63（98.4） | 24(72.7) | 10.070 | 0.105 |
|  | 丧偶 | 7（4.5） | 3(4.95) | 2（3.1） | 2(6.1) |  |  |
|  | 离异 | 18（11.6） | 6(11.5) | 7（10.9） | 5(15.2) |  |  |
| 文化程度 | 小学及以下 | 25（16.1） | 9(15.6) | 7（10.9） | 9(27.3) | 9.655 | 0.085^*^ |
|  | 初中 | 62（40） | 23(40.2) | 19（29.7） | 20(60.6) |  |  |
|  | 高中/专科 | 51（32.9） | 18(32.8) | 29（45.3） | 4(12.1) |  |  |
|  | 大学及以上 | 17（11） | 8(11.5) | 9（14.1） | 0 |  |  |
| 工作状态 | 离退休 | 144（92.9） | 54(92.6) | 59（92.2） | 31(93.9) | 16.857 | 0.169 |
|  | 其他 | 11（7.1） | 4(7.4) | 5（7.8） | 2(6.1) |  |  |
| 月收入 | ＜1000 | 6（3.9） | 2(4.1) | 3（4.7） | 1(3) | 30.047 | 0.881^*^ |
|  | 1000—3000 | 27（17.4） | 10(17.2) | 11（17.2） | 6(18.2) |  |  |
|  | 3000—5000 | 58（37.4） | 21(36.9) | 23（35.9） | 14(42.4) |  |  |
|  | ＞5000 | 64（41.3） | 20(34.4) | 31（48.4） | 13(39.4) |  |  |
| 医疗方式 | 新农合 | 21（13.5） | 8(13.1) | 8（12.5） | 5(15.2) | 17.398 | 0.194^*^ |
|  | 医疗保险 | 126（81.3） | 46(80.3) | 55（85.9） | 25(75.8) |  |  |
|  | 公费医疗 | 3（1.9） | 1(1.6) | 1（1.6） | 1(3) |  |  |
|  | 自费 | 5（3.2） | 3(4.9) | 0 | 2(6.1) |  |  |
| CCI分级 | 低度（2—3分） | 13（8.5） | 5(8.2) | 8（12.5） | 0 | 9.211 | 0.077^*^ |
|  | 中度（4—5分） | 61（39.4） | 23(40.2) | 25（39.1） | 13(39.4) |  |  |
|  | 重度（≥6分） | 81（52.3） | 30(51.6) | 31（48.4） | 20(60.6) |  |  |

表2 纳入研究对象的ADL、NRS 2002、SATI资料

| 项目 | 频率[例] | 最大值 | 最小值 | 中位数 | 四分位间距（P_25_，P_75_） |
| --- | --- | --- | --- | --- | --- |
| 日常活动能力T1 | 155 | 100 | 55 | 100 | 90，100 |
| 日常活动能力T2 | 155 | 100 | 10 | 90 | 85，95 |
| 日常活动能力 T3 | 155 | 100 | 0 | 95 | 90，100 |
| 营养风T1 | 155 | 5 | 1 | 2 | 2，3 |
| 营养风险T2 | 155 | 6 | 2 | 3 | 3，4 |
| 营养风险T3 | 155 | 7 | 1 | 3 | 2，4 |
| 特质焦虑 | 155 | 50 | 20 | 32 | 29，35 |
| 状态焦虑T1 | 155 | 60 | 28 | 43 | 38，47 |
| 状态焦虑T2 | 155 | 59 | 24 | 38 | 35，43 |
| 状态焦虑 T3 | 155 | 59 | 22 | 35 | 32，40 |

表3 纳入研究对象的ADL、NRS 2002、SATI的多重比较

|  | F | P | 多重比较 |
| --- | --- | --- | --- |
| 日常活动能力 | 35.019 | ＜0.001 | T1＞T3＞T2 |
| 营养风险 | 21.597 | .003 | T1＜T3＜T2 |
| 状态-特质焦虑 | 66.393 | ＜0.001 | T1＞T2＞T3＞N |

注：N为特质焦虑状况

表4 衰弱发展变化模型潜在类别增长模型拟合结果

| 模型 | LL | AIC | BIC | aBIC | Entropy | BLRTP | VLMRP | 类别比例 |
| --- | --- | --- | --- | --- | --- | --- | --- | --- |
| 1 | -1405.227 | 2844.453 | 2896.081 | 2842.274 | — | — | — | — |
| 2 | -1395.946 | 2847.892 | 2932.927 | 2844.303 | 0.734 | ＜0.001 | 0.4494 | 77.9/22.1 |
| 3 | -1390.582 | 2855.892 | 2853.075 | 2851.790 | 0.832 | ＜0.001 | 0.4315 | 63.6/29.9/6.5 |
| 4 | -1388.423 | 2848.846 | 2958.176 | 2844.230 | 0.860 | ＜0.001 | 0.2398 | 29.9/22.1/5.2/42.8 |
| 5 | -1384.177 | 2848.355 | 2969.833 | 2843.227 | 0.859 | 0.030 | 0.2204 | 29.9/22.1/5.2/42.9/0 |

表5 衰弱发展变化不同模型参数估计

| 类别 | Intercept | Slope | Quadratic |
| --- | --- | --- | --- |
|  | M(SE) | M(SE) | M(SE) |
| 1 | 3.523（0.061） | 0.608（＜0.001）*** | -3.253（＜0.001）*** |
| 2 | 0.236（0.018）* | -4.038（＜0.001）*** | -4.434（＜0.001）*** |
| 3 | 4.603（＜0.001）*** | -1.803（＜0.001）*** | 2.094（＜0.001）*** |
| 4 | 0.267（0.046）* | 3.713（＜0.001）*** | 3.952（＜0.001）*** |

表6 患者T1—T3时期衰弱发生状况

| 模型分类 | T1—T2 | T2—T3 | T1—T3 |
| --- | --- | --- | --- |
| 无衰弱组（n=42） | 3.696 | -5.831 | 6.370 |
| 状态加重组（n=49） | .137** | 1.052 | .309*** |
| 状态改善组（n=34） | -1.133 | -.084* | -.221** |
| 持续衰弱组（n=30） | 2.577 | 4.258 | 4.936 |

表7 一般人口学因素对衰弱发展变化趋势的单因素分析

| 项目 | 分类 | 无衰弱组（n=21） | 状态加重组（n=62） | 持续衰弱组（n=41） | 状态改善组（n=31） | X^2^ | P |
| --- | --- | --- | --- | --- | --- | --- | --- |
| 性别 | 男 | 9（47.6） | 32(51) | 19（46.7） | 16（50） | 5.905 | .052 |
|  | 女 | 11（52.4） | 30(49) | 22（53.3） | 15（50） |  |  |
| 年龄 | ＜65岁 | 10(50) | 0 | 11(26.7) | 17(58.8) | 50.128^*^ | .000 |
|  | 65—74岁 | 7(28.6) | 40(65.3) | 19(46.7) | 10(32.4) |  |  |
|  | 75—84岁 | 4(21.4) | 17(28.6) | 9(23.3) | 4(8.8) |  |  |
|  | ≥85岁 | 0 | 4（6.1） | 2(13.3) | 0 |  |  |
| BMI（Kg/m2） | ＜18.5 | 1（4.8） | 11（18.5） | 1（3.3） | 1（2.9） | 73.965^*^ | .000 |
|  | 18.5—23.9 | 17（81） | 16（24.5） | 15（36.7） | 16（55.9） |  |  |
|  | 24—27.9 | 2（11.9） | 15（34.6） | 14（33.3） | 12（32.4） |  |  |
|  | ≥28 | 1(2.4) | 21（22.4） | 11（26.7） | 2（8.8） |  |  |
| 吸烟史 | 无 | 13（54.8） | 48(75.5) | 30（73.3） | 23（76.5） | 4.683^*^ | .321 |
|  | 有 | 4（11.9） | 7(12.2) | 5（13.3） | 4（11.8） |  |  |
|  | 曾经吸烟 | 4（11.9） | 7(12.2) | 6（13.3） | 4（11.8） |  |  |
| 饮酒史 | 无 | 15（73.8） | 47(73.5) | 30（73.3） | 22（73.5） | 4.581^*^ | .333 |
|  | 有 | 4（19） | 11(19.4) | 8（20） | 6（20.6） |  |  |
|  | 曾经饮酒 | 2（7.1） | 3(6.1) | 3（6.7） | 3（5.9） |  |  |
| 居住地 | 城镇 | 17（81） | 49(79.6) | 32（80） | 25（79.4） | .694 | .707 |
|  | 农村 | 4（19） | 13(20.4) | 9（20） | 6（20.6） |  |  |
| 居住状况 | 独居 | 1（2.4） | 3(6.1) | 5（13.3） | 1（2.9） | 1.438^*^ | .384 |
|  | 与配偶居住 | 12（57.1） | 10(18.4) | 14（33.3） | 19（61.8） |  |  |
|  | 与子女居住 | 8（40.5） | 48(75.5) | 20（50） | 21（35.3） |  |  |
|  | 其他 | 0 | 0 | 2（3） | 0 |  |  |
| 婚姻状况 | 已婚 | 16（83.3） | 52(83.7) | 35（83.3） | 26（85.3） | 8.979^*^ | .062 |
|  | 丧偶 | 1（4.8） | 2(4.1) | 1（3） | 2（5.9） |  |  |
|  | 离异 | 4（11.9） | 7(12.2) | 5（13.3） | 3（8.8） |  |  |
| 文化程度 | 小学及以下 | 1（2.4） | 16(26.5) | 0 | 1（2.9） | 13.237^*^ | .039 |
|  | 初中 | 5（26.2） | 31(51) | 34（83.3） | 4（11.8） |  |  |
|  | 高中/专科 | 9（45.2） | 15(22.4) | 6（13.3） | 21（70.1） |  |  |
|  | 大学及以上 | 56（26.2） | 0 | 1（3.1） | 5（14.7） |  |  |
| 月收入 | ＜1000 | 1（4.8） | 2(4.1) | 1（3） | 1（2.9） | 4.324^*^ | .633 |
|  | 1000—3000 | 5（19.3） | 11(18.4) | 7（16.7） | 6（17.6） |  |  |
|  | 3000—5000 | 7（35.7） | 23(36.7) | 15（36.7） | 12（38.2） |  |  |
|  | ＞5000 | 8（40.2） | 26(40.8) | 19（43.3） | 13（41.2） |  |  |
| 医疗方式 | 新农合 | 3（14.3） | 10（14.3) | 6（13.3） | 5（14.7） | 6.199^*^ | .401 |
|  | 医疗保险 | 16（81） | 50(81.6) | 33（80） | 25（82.3） |  |  |
|  | 公费医疗 | 1（2.4） | 0 | 1（3） | 0 |  |  |
|  | 自费 | 1（2.4） | 2(4.1) | 1（3） | 1(2.9) |  |  |
| CCI | 低度（2—3分） | 5（21.4） | 0 | 0 | 4(11.8) | 55.629^*^ | .000 |
|  | 中度（4—5分） | 10（47.6） | 15(24.5) | 3（6.7） | 24(79.4) |  |  |
|  | 重度（≥6分） | 6（31） | 47(75.5) | 38（93.3） | 3(8.8) |  |  |

表8 手术相关因素对不同类别衰弱发展变化的单因素分析

| 项目 | 分类 | 无衰弱组（n=21） | 状态加重组（n=62） | 状态改善组（n=31） | 持续衰弱组（n=41） | X^2^ | P值 |
| --- | --- | --- | --- | --- | --- | --- | --- |
| 手术等级 | 三级 | 7(31) | 51(81.6) | 22(70.6) | 13(30) | 3.610 | 1.037 |
|  | 四级 | 14(69) | 11(18.4) | 9(29.4) | 28(70) |  |  |
| 手术种类 | 胆囊切除术 | 7(35.7) | 6(10.2) | 15(47.1) | 0 | 21.204^*^ | .002 |
|  | 胆管/道取石术 | 4(19) | 3(4.1) | 2(7.1) | 0 |  |  |
|  | 肝（部分）切除术 | 4(19) | 1(2) | 4（11.9) | 1(3) |  |  |
|  | 低位直肠前切术 | 5(23.8) | 3(4.1) | 4(14.3) | 1(3) |  |  |
|  | 结直肠根治术 | 1(4.8) | 15(24.5) | 3(8.9) | 7(16.7) |  |  |
|  | 胰腺坏死组织清除术 | 0 | 22(34.7) | 0 | 17(40) |  |  |
|  | 胃部分切除术 | 0 | 13(20.4) | 1(2.9) | 15(36.7) |  |  |
| 手术时间 | ≤1h | 14(69) | 4(6.1) | 20(64.7) | 0 | 1.524^*^ | .158 |
|  | 1—4h（不包括4h） | 7(31) | 13(20.4) | 9(29.4) | 15(36.7) |  |  |
|  | 4—7h（不包括7h） | 0 | 32(51) | 2(5.9) | 22(53.3) |  |  |
|  | ≥8h | 0 | 6(10) | 0 | 4(10) |  |  |
| 术前疼痛 | 无疼痛（0） | 2(11.9) | 6(10) | 4(11.8) | 4(10) | 2.429^*^ | .057 |
|  | 轻度疼痛（1—3分） | 15(71.4) | 43(69.4) | 22(70.6) | 28(70) |  |  |
|  | 中度疼痛（4—6分） | 4(16.7) | 11(18.4) | 4(11.8) | 9(20) |  |  |
|  | 重度疼痛（7—10分） | 0 | 1(2) | 0 | 0 |  |  |
| 术前胃管 | 无 | 20(95.2) | 37(59.2) | 30(97.1) | 8(20) | 10.309^*^ | .006 |
|  | 有 | 1(4.8) | 25(40.8) | 1(2.9) | 33(80) |  |  |
| 术前尿管 | 无 | 21(100) | 48(77.6) | 31(100) | 21(73.3) | 8.461^*^ | .015 |
|  | 有 | 0 | 14(22.4) | 0 | 15（26.7) |  |  |
| 术中出血量（ml） | ＜50 | 19(88.1) | 4(6.1) | 27(88.2) | 0 | 4.605^*^ | .237 |
|  | 50—99 | 2(11.9) | 32(51) | 3(8.9) | 2(6.7) |  |  |
|  | 100—149 | 0 | 10(16.3) | 1(2.9) | 14(33.3) |  |  |
|  | 150—199 | 0 | 8(12.2) | 0 | 7(16.7) |  |  |
|  | ≥200 | 0 | 9(14.3) | 0 | 18(43.3) |  |  |
| 术中留置腹腔引流管 | 无 | 19(88.1) | 19(30.6) | 26(82.3) | 1(3.3) | 38.351^*^ | .000 |
|  | 有 | 2(11.9) | 43(69.4) | 5(17.6) | 40(96.7) |  |  |
| 引流管留置时间 | 少于1周 | 20(97.6) | 49(79.6) | 31(100) | 32(76.7) | 41.423^*^ | .057 |
|  | 1—2周 | 1(2.4) | 9(14.3) | 0 | 4(10) |  |  |
|  | 超过2周 | 0 | 4(6.1) | 0 | 5（13.3) |  |  |
| 有无进入ICU | 无 | 20(97.6) | 30(49) | 30(97.1) | 12(30) | 53.516^*^ | .000 |
|  | 是 | 1(2.4) | 32(51) | 1(2.9) | 29(70) |  |  |
| ICU入住时间 | 1—3天 | 6(26.2) | 1(2) | 8(26.5) | 1(3.3) | 54.084^*^ | .000 |
|  | 4—6天 | 1(4.8) | 6(10.2) | 1(2.9) | 14(36.6) |  |  |
|  | 7—10天 | 0 | 1(2) | 0 | 3(6.7) |  |  |
|  | 大于10天 | 0 | 3(4.1) | 0 | 3(6.7) |  |  |
| 术后24h疼痛 | 轻度（1—3分） | 1(7.1) | 0 | 1(2.9) | 0 | 4.462^*^ | .064 |
|  | 中度（4—6分） | 20(92.9) | 34(55.1) | 30(97.1) | 7(16.7) |  |  |
|  | 重度（7—10分） | 0 | 28(44.9) | 0 | 34(83.3) |  |  |
| 术后镇痛/镇静药物使用 | 无 | 17(81) | 13(20.4) | 26(82.3) | 7（16.7） | 36.616 | .000 |
|  | 有 | 4(19) | 49(79.6) | 5(17.6) | 34（83.3） |  |  |
| 术后首次下床时间 | 1—3天 | 21（100) | 39(63.3) | 31(100) | 16（40） | 54.723^*^ | .000 |
|  | 4—6天 | 0 | 20(32.7) | 0 | 20（50） |  |  |
|  | ≥7天 | 0 | 3(4.1) | 0 | 5（10） |  |  |
| 术后首次排气时间 | 1—3天 | 21(100) | 56(89.8) | 31(100) | 24（60） | 2.694^*^ | .408 |
|  | 4—6天 | 0 | 6(10.2) | 0 | 16（36.6） |  |  |
|  | ≥7天 | 0 | 0 | 0 | 1（3.3） |  |  |
| 术后首次经口进食时间 | 1—3天 | 20(97.6) | 40(65.3) | 30(97.1) | 11（26.6） | 55.245^*^ | .000 |
|  | 4—6天 | 1(2.4) | 22(34.7) | 1(2.9) | 28（70） |  |  |
|  | ≥7天 | 0 | 0 | 0 | 1（3.3） |  |  |
| 术后并发症 | 无 | 12(69) | 5(8.2) | 13（41.2) | 0 | 67.638^*^ | .000 |
|  | Ⅰ级 | 4(19) | 19(30.6) | 14(44.1) | 4（10） |  |  |
|  | Ⅱ级 | 1(7.1) | 13(20.4) | 4(11.8) | 8（20） |  |  |
|  | Ⅲ级 | 1(2.4) | 4(6.1) | 0 | 7（16.7） |  |  |
|  | Ⅳ级 | 1（2.4） | 20(32.7) | 1(2.9) | 21（50） |  |  |
|  | Ⅴ级 | 0 | 1(2) | 0 | 1（3.3） |  |  |

表9 日常活动能力、营养、焦虑在不同类别衰弱发展变化的单因素分析

| 项目 | 组别 | 频数（例） | 均值±标准差（MD） | F | P |
| --- | --- | --- | --- | --- | --- |
| 日常活动能力T1 | 无衰弱组 | 21 | 96.72±0.530 | 32.019 | .000 |
|  | 状态加重组 | 62 | 93.56±0.648 |  |  |
|  | 持续衰弱组 | 41 | 91.03±1.702 |  |  |
|  | 状态改善组 | 31 | 94.53±0.819 |  |  |
| 日常活动能力T2 | 无衰弱组 | 21 | 93.51±2.018 | 25.748 | .000 |
|  | 状态加重组 | 62 | 84.93±1.530 |  |  |
|  | 持续衰弱组 | 41 | 85.47±1.018 |  |  |
|  | 状态改善组 | 31 | 91.47±1.839 |  |  |
| 日常活动能力T3 | 无衰弱组 | 21 | 97.36±0.507 | 27.143 | .000 |
|  | 状态加重组 | 62 | 85.96±0.279 |  |  |
|  | 持续衰弱组 | 41 | 88.19±0.994 |  |  |
|  | 状态改善组 | 31 | 96.07±0.148 |  |  |
| 营养风险T1 | 无衰弱组 | 21 | 2.08±0.039 | 11.626 | .000 |
|  | 状态加重组 | 62 | 2.18±0.073 |  |  |
|  | 持续衰弱组 | 41 | 3.34±1.609 |  |  |
|  | 状态改善组 | 31 | 3.04±0.983 |  |  |
| 营养风险T2 | 无衰弱组 | 21 | 2.05±2.519 | 15.379 | .000 |
|  | 状态加重组 | 62 | 3.72±1.174 |  |  |
|  | 持续衰弱组 | 41 | 3.72±1.174 |  |  |
|  | 状态改善组 | 31 | 3.04±0.983 |  |  |
| 营养风险T3 | 无衰弱组 | 21 | 2.11±0.586 | 12.987 | .000 |
|  | 状态加重组 | 62 | 3.80±1.458 |  |  |
|  | 持续衰弱组 | 41 | 3.25±0.154 |  |  |
|  | 状态改善组 | 31 | 2.89±0.105 |  |  |
| 特质焦虑 | 无衰弱组 | 21 | 29.38±0.753 | 8.442 | .047 |
|  | 状态加重组 | 62 | 31.48±1.024 |  |  |
|  | 持续衰弱组 | 41 | 32.24±0.427 |  |  |
|  | 状态改善组 | 31 | 28.76±0.507 |  |  |
| 状态焦虑T1 | 无衰弱组 | 21 | 38.57±0.651 | 3.393 | .092 |
|  | 状态加重组 | 62 | 39.95±1.753 |  |  |
|  | 持续衰弱组 | 41 | 40.03±1.466 |  |  |
|  | 状态改善组 | 31 | 37.80±0.941 |  |  |
| 状态焦虑T2 | 无衰弱组 | 21 | 34.47±0.938 | 7.298 | .001 |
|  | 状态加重组 | 62 | 42.55±0.798 |  |  |
|  | 持续衰弱组 | 41 | 43.16±0.498 |  |  |
|  | 状态改善组 | 31 | 30.65±1.278 |  |  |
| 状态焦虑T3 | 无衰弱组 | 21 | 32.72±0.897 | 10.372 | .000 |
|  | 状态加重组 | 62 | 42.50±1.087 |  |  |
|  | 持续衰弱组 | 41 | 41.95±1.205 |  |  |
|  | 状态改善组 | 31 | 30.39±0.635 |  |  |

表10 哑变量赋值情况

| 变量名称 | | 赋值 |
| --- | --- | --- |
| 因变量 | 衰弱分组模型 | 无衰弱组=0，持续衰弱组=1，状态加重组=2，状态改善组=3 |
| 自变量 | 年龄 | ＜65=0，65-74=1，75-84=2，≥85=3 |
|  | BMI | ＜18.5=0，18.5—23.9=1，24—27.9=2，≥28=3 |
|  | 文化程度 | 小学及以下=1，初中=2，高中/专科=3，大学及以上=4 |
|  | CCI分级 | 低度（2—3）=1，中度（4—5）=2，重度（≥6）=3 |
|  | 手术种类 | 胆囊切除术=1，胆管/道取石术=2，肝（部分）切除术=3，低位直肠前切术=4，结直肠根治术=5，胰腺坏死组织清除术=6，胃部分切除术=7 |
|  | 有无留置引流管 | 无=0，有=1 |
|  | 术中留置引流时间 | 少于1周=0，1—2周=1，超过2周=2 |
|  | 有无进入ICU | 无=0，有=1 |
|  | 术后有无镇静/镇痛药物使用 | 无=0，有=1 |
|  | 首次下床/排气进食时间 | 1—3天=1，4—6天=2，≥7=3 |
|  | 术后并发症 | 不足Ⅱ级=0，Ⅱ级=1，Ⅲ级=2，Ⅳ级=3，Ⅴ级=4 |
|  | 日常活动能力 | 输入数值变量 |
|  | 营养风险 | 输入数值变量 |
|  | 焦虑情绪 | 输入数值变量 |

表11 不同类别衰弱发展变化的多因素分析

| 组别 | 变量 | B | SE | Wald | P值 | Exp（B） | 95%CI |
| --- | --- | --- | --- | --- | --- | --- | --- |
| 状态加重组 | BMI＜18.5（kg/m^2^） | 2.656 | .695 | 14.617 | .000 | 3.035 | [1.259-5.572] |
|  | BMI≥28（kg/m^2^） | .415 | .080 | 8.537 | .000 | 4.240 | [1.649-7.572] |
|  | CCI分级重度  （≥6分） | 1.253 | 1.077 | 1.100 | .029 | 3.745 | [2.116-5.775] |
|  | 胃部分切除术 | 1.217 | .830 | .200 | .000 | 4.410 | [3.242-6.767] |
|  | 术中留置腹腔引流管 | 1.952 | .820 | 5.668 | .001 | 5.502 | [3. 559-7.345] |
|  | 术后首次下床时间（4—6天) | .968 | .335 | 8.357 | .004 | 2.475 | [1.312-4.624] |
|  | 术后镇痛/镇静药物使用 | 2.981 | .077 | 15.690 | .013 | 2.357 | [1.011-3.972] |
|  | 术后并发症（Ⅱ级） | 1.002 | 1.954 | 94.532 | .000 | 4.851 | [2.832--7.171] |
|  | 日常活动能力T2 | -1.812 | .877 | 4.268 | .009 | 0.196 | [0.093-0.246] |
|  | 营养风险T2 | .193 | .037 | 27.349 | .000 | 3.197 | [1.050-6.384] |
|  | 状态焦虑T2 | .150 | .051 | 8.537 | .003 | 2.702 | [1.259-4.572] |
| 持续衰弱组 | BMI＜18.5（kg/m^2^） | 9.771 | 4.018 | 67.295 | .012 | 3.257 | [1.028-5.590] |
|  | BMI≥28（kg/m^2^） | 8.370 | .086 | 17.189 | .000 | 3.047 | [1.003-6.003] |
|  | CCI分级重度（≥6分） | 5.898 | 1.008 | .311 | .020 | 4.052 | [2.466-5.135] |
|  | 胰腺坏死组织清除术 | 1.341 | .804 | 3.919 | .000 | 3.179 | [1.214-4.088] |
|  | 胃部分切除术 | 1.476 | 1.758 | 83.985 | .000 | 4.063 | [2.503-6.821] |
|  | 术中留置腹腔引流管 | 3.528 | .643 | 10.998 | .029 | 2.327 | [1.382-3.668] |
|  | 引流时间（＞2周） | 5.178 | .437 | 2.099 | .001 | 3.009 | [1.350-5.071] |
|  | 术后首次下床时间4—6天 | 1.118 | .678 | .006 | .011 | 3.760 | [2.681-5.194] |
|  | 术后镇痛/镇静药物使用 | 1.993 | .796 | 2.531 | .001 | 5.013 | [3.138-7.790] |
|  | 术后并发症（Ⅳ级） | 2.052 | .989 | 7.484 | .026 | 1.640 | [1.023-4.121] |
|  | 日常活动能力T1 | -8.594 | .143 | 4.641 | .000 | 0.501 | [0.267-0.913] |
|  | 营养风险T1 | 7.033 | .001 | .000 | .000 | 3.015 | [1.138-6.223] |
|  | 特质焦虑 | 1.752 | .664 | 3.084 | .003 | 4.003 | [2.500-6.926] |
| 状态改善组 | BMI：  18.5—23.9kg/m2 | -4.233 | 1.184 | 8.551 | .003 | 3.481 | [1.054-1.913] |
|  | 胆囊切除术 | -.728 | .655 | 1.236 | .000 | 5.035 | [2.012-6.556] |
|  | 低位直肠切除术 | -1.542 | .652 | 5.590 | .008 | 4.024 | [1.820-4.264] |
|  | 术后首次下床时间（1-3天） | -.744 | .767 | 1.940 | .002 | 0.361 | [0.108-0.760] |
|  | 术后首次进食时间（1-3天） | -1.097 | 3.338 | 1.108 | .001 | 0.420 | [0.39-0.645] |
|  | 术后并发症  （不足Ⅱ级） | -1.372 | .639 | 4.610 | .032 | 0.351 | [0.120-0.624] |
|  | 活动能力（T2） | -.562 | 1.008 | 1.311 | .000 | 5.017 | [1.538-5.414] |
|  | 营养风险（T1） | 9.002 | 1.954 | 4.532 | .000 | 0.479 | [0.332--0.871] |
|  | 营养风险（T2） | .356 | .086 | 3.189 | .000 | 0.361 | [0.188--0.524] |
|  | 状态焦虑（T1） | 3.162 | 1.194 | 2.008 | .008 | 0.649 | [0.503--0.821] |
