# Supplementary material for: Frailty trajectory and its associated factors in older patients undergoing abdominal surgery involving the digestive system: A longitudinal study
Source: PLoS One. 2025 Aug 8;20(8):e0330093. doi: 10.1371/journal.pone.0330093 (PMC12334016; doi:10.1371/journal.pone.0330093)
Supplement: Attachment — Submitted filename: Response to Reviewers.docx [file pone.0330093.s003.docx]

**Response to Reviewers**

Dear reviewer,

Thank you very much for your comments and professional advice. These opinions help to lmprove academic rigor of our article Based on your suggestion and request,we have made corrected modifications on the revised manuscript. Meanwhile. The manuscript had be reviewed and edited by language services of AJE.We hope that our work can be improved again.Furthermore,we would like to show the details as follows:

Reviewer #1: Reviewer Comments

Major Concerns
1. Study Population Age Definition (Why ≥60 years?)
The rationale for setting the inclusion criterion at age 60 rather than the more commonly used threshold of 65 years is unclear. A justification should be provided, especially given that frailty-related literature often focuses on populations ≥65 years.
**Response 1**

The-author's answer:The reasons for setting the inclusion criteria for the study population at ≥60 years old are as follows: On the one hand, the WHO defines the age of elderly individuals in developing countries as ≥60 years, and China's definition and regulations for the elderly also specify ≥60 years; additionally, this study considers that frailty is a non-specific state in older adults characterized by increased physical vulnerability and reduced stress resistance due to decreased physiological reserve. Such individuals are more significantly affected by grade III and IV major surgeries and more likely to develop frailty. Therefore, the population was defined as ≥60 years old based on the above reasons.
2. Albumin and Other Nutritional Markers
The study discusses nutritional status, yet serum albumin, total protein, and prealbumin—commonly used clinical markers—are not included as explanatory variables. Were these data unavailable, or were they excluded for another reason? Please clarify.
**Response 2**

The-author's answer:In the discussion of nutritional status, this study only used the NRS 2002 scale, and commonly used clinical indicators (e.g., serum albumin, total protein, and prealbumin) were not included as explanatory variables. This is primarily due to limitations related to the study location and time: the hospital where the study was conducted did not require all surgical patients to undergo the laboratory tests required by the study between 2023 and 2024, resulting in the study being unable to accurately obtain relevant data for each patient. Therefore, after discussion by the research team, a professional scale for nutritional risk assessment was selected, which also constitutes one of the limitations of the study. Moving forward, we will also carry out multidisciplinary collaboration to further improve the in-depth analysis of nutritional status.
3. Surgical Approach Misrepresentation
Table 1 lists “laparoscopy: 100%, laparotomy: 0%,” which is inconsistent with the types of procedures included (e.g., gastrectomy, pancreaticoduodenectomy), many of which are still commonly performed via open surgery. This discrepancy must be clarified. Actual surgical approach data (laparoscopic vs open) should be accurately reported.
**Response 3**

The-author's answer:

The We re-investigated the original medical records of the study participants to verify the surgical approach (laparotomy vs. laparoscopy), clarified the inaccurate data in the report, and made corrections (laparoscopy: 66.45% vs. laparotomy: 33.55%).
4. Poor Table Design and Lack of Definitions
Many of the tables are difficult to interpret. Variable names are inconsistently labeled or undefined (e.g., “income,” “gastric tube”). Some variables appear more than once (e.g., “preoperative gastric tube”), and the definitions of categorical cutoffs (e.g., “bed time,” “drainage”) are not provided. Without clear definitions, clinical interpretation is impaired. Please reformat the tables for clarity and include a variable definitions table or footnotes.
**Response 4**

The-author's answer:Thanks for your suggestion.However, We have rechecked data related to study participants and revised the table content, including standardizing variable names, clarifying their specific definitions, verifying the data, adding footnotes, and other adjustments. Details are presented in the table. And we hope the revised manuscript could be acceptable for you.

Minor Concerns

1. Placement of Table 3 (Model Fitting Table)

Table 3 presents technical statistics (AIC, BIC, entropy, BLRT) that may not be accessible or meaningful to general surgical readers. Since it does not contribute directly to the clinical narrative, I suggest moving Table 3 to the supplementary material and focusing the main text on clinically interpretable content such as Figure 2–5.

The-author's answer:We think this is an excellent suggestion. We have adjusted the position of the table based on your suggestions, so that it is now presented as supplementary material.

2. “One in Ten” Rule in Multivariable Models

The multivariable analyses shown in Figures 3–5 include many predictors relative to the group sizes (e.g., 9 predictors in the improvement group with only 21 patients). This may violate the "one-in-ten rule" and lead to overfitting. The authors should clarify how variables were selected and consider model simplification or penalized regression techniques if appropriate.

The-author's answer:Thank you for your valuable suggestions. In the multivariate analysis, we incorporated the multivariate model analysis based on the results of the univariate analysis. This may violate the "one-in-ten rule" and lead to overfitting. After group discussion, simplifying the model might affect the conclusions of this study. Therefore, we plan to further include more study subjects, expand the sample size, and conduct in-depth analysis of the influencing factors in subsequent research.

3. Typographical and Formatting Issues

There are minor inconsistencies in table formatting (e.g., alignment, repeated rows) that reduce readability and should be addressed during revision.

The-author's answer:Thanks for your careful checks. We are sorry for our carelessness. Based

on your comments, we have revised the typesetting and formatting issues, especially the tables.

**Reviewer #2: Dear Editor,**

1. Whether or not chemotherapy is administered after surgery can significantly impact the manifestation of frailty, as certain chemotherapy drugs can adversely affect physical condition and lead to frailty. Why did the author fail to include chemotherapy as an associated factor?

**Response 1**

The-author's answer:This study primarily focused on the impact of abdominal surgery-related factors on frailty in elderly patients, with a follow-up period extending to 1 month postoperatively. Although chemotherapy, as one of the adjuvant therapies for cancer patients, may lead to decline in physical function and frailty, it was not included in the primary analytical dimension after comprehensively considering its limited relevance to the research objective.

2. The surgical spot will influence the timing of the first postoperative meal and the placement of a peritoneal drainage tube. In other words, there is multicollinearity among these three factors. If the authors include all three factors in the multivariate analysis simultaneously, it will lead to greater errors and increase the instability of parameter estimation. How do the authors explain or avoid this influence?

**Response 2**

The-author's answer:Before conducting multivariate logistic regression analysis, the study first performed univariate analysis on all variables (including surgical site, timing of the first postoperative meal, and placement of peritoneal drainage tube). Independent influencing factors were screened, and only variables with statistical significance (P<0.05) in the univariate analysis were included in the final multivariate model. This screening process effectively eliminates highly correlated variables, avoids simultaneously including factors with multicollinearity, and thereby reduces model errors and the instability of parameter estimation. Additionally, the surgical sites involved in this study included the gastrointestinal tract, hepatobiliary system, and pancreas. In the preliminary literature review, no significant evidence was found to confirm obvious multicollinearity among surgical site, timing of the first postoperative meal, and peritoneal drainage tube placement. Of course, we have deeply reflected on and discussed the issue raised by the expert. This aspect was not considered in the preliminary research, and we will conduct in-depth studies to further explore how to minimize such errors and enhance the stability of parameters.

3. In Figs. 3 and 4, the OR value of ADL is less than 0, while that of NRS2002 and STAI is are greater than 0. In Fig. 5, the above three values are reversed, while other factors such as bed time and complications are all greater than 0. The authors should provide an explanation for these discrepancies in the discussion section.

**Response 3**

The-author's answer:Thank you for carefully reading the article and putting forward some valuable suggestions. In response to the issues you raised, we conducted a discussion among the research team and revised the errors. The details are as follows:

The We re-reviewed the original data, conducted statistical analysis and statistical testing, and have revised the OR values in Figures 3 and 4: An OR value <1 indicates a protective factor for frailty status (worsening, persistence, improvement); an OR value >1 indicates an exposure factor, and supplementary explanations have been added in the discussion section.

4. Many of the data presented in the manuscript are inconsistent and confusing:

1) In table 1, the number of smokers, education level, monthly income, and CCI are not equal to the total number (155).

2) In Table 3, the BIC of four-category model is higher than that of three-category model, however the description indicates the opposite.

3) The content described in the results shows “There were 41 patients in class 3, accounting for 26.7% of the total.” However, in Table 2, the frailty was only 33 before surgery (T1).

4) The numbers for the "no frailty" and "frailty improvement" in Table 4 are inconsistent with those presented in the preceding text. Additionally, there is a typographical error in the male column* frailty improvement" in Table 4. Furthermore, there are problems with the data of BMI*frailty exacerbation, Age*persistent frailty, Drinking status*frailty exacerbation, Habitation status*frailty exacerbation, persistent frailty, frailty improvement, Education level*no frailty, Monthly income*persistent frailty, frailty improvement.

5) The data of Surgical spot* frailty exacerbation, frailty improvement，Preoperative pain* frailty exacerbation, frailty improvement，Preoperative urinary catheter* persistent frailty，Postoperative complications* no frailty, persistent frailty in Table 5 are problematic.

6) In the results section, should the second line of the "Frailty trajectories" section have "table2" instead of "table3"?

**Response 4**

The-author's answer:We were really sorry for our careless mistakes.Thank you for yourreminder.

1) We have re-reviewed the original data, revised the values of the number of smokers, education level, monthly income, and Charlson Comorbidity Index (CCI),
2) We have reconducted statistical calculations to adjust the Bayesian Information Criterion (BIC) value of the four-category model.
3) We re-analyzed and recalculated the original data of the study subjects and revised Data in Table 2.
4) Data in Table 4 have been revised.
5) Data in Table 5 have been revised.
6) Table 2 has been revised to Table 3 in "Frailty trajectories" section.

**Thank you very much for your attention and time.Look for ward to-hearing from you.**

**Yours sincerely,**

Xinran Wang
